# Assessment of a porcine circovirus type 2 vaccine prototype through anatomopathological analysis and its correlation with blood viral load

**DOI:** 10.3389/fvets.2025.1656345

**Published:** 2026-01-02

**Authors:** Fernanda Larenas-Muñoz, Paloma De Barbieri, César Mellado, Fátima Reyes, Jorge Toledo, Víctor Neira, Emilio Lamazares, Alvaro Ruiz-Garrido

**Affiliations:** 1 Departamento de Patología y Medicina Preventiva, Facultad de Ciencias Veterinarias, Universidad de Concepción, Chillán, Chile; 2 Departamento de Farmacología, Facultad de Ciencias Biológicas, Universidad de Concepción, Concepción, Chile; 3 Laboratorio de Biotecnología y Biofármacos, Departamento de Fisiopatología, Facultad de Ciencias Biológicas, Universidad de Concepción, Concepción, Chile; 4 Departamento de Medicina Preventiva Animal, Facultad de Ciencias Veterinarias y Pecuarias, Universidad de Chile, Santiago, Chile

**Keywords:** PCV2, porcine circovirus, pigs, pathogenesis, viremia, PCVAD

## Abstract

Porcine circovirus type 2 (PCV2) is an endemic pathogen of global relevance, responsible for porcine circovirus-associated diseases (PCVAD). Consequently, vaccination against PCV2 is a standard practice in intensive swine production systems. This study aimed to evaluate the effect of a prototype vaccine on PCV2 viral load and associated lesions. Thirty-nine pigs from a high-health-status farm were randomly assigned to three experimental groups (*n* = 13 per group), corresponding to a placebo group, a commercial vaccine group and a prototype vaccine group. Viral load was assessed by qPCR from serum samples, while lesions were evaluated through necropsy and histopathological analysis of lymph nodes and lungs tissue. No statistically significant differences in viral loads were observed among the three groups, and most animals did not exhibit detectable viremia. However, the placebo group showed more numerous and severe lesions in lymph nodes and lungs compared to the vaccinated groups, with the commercial vaccine group showing milder lesions than the prototype vaccine group. These findings suggest that the absence of viremia in most animals may reflect the timing of infection or effective containment by host immunity. Both the commercial and prototype vaccines were associated with reduced lesion severity, although the prototype vaccine demonstrated an intermediate performance between the placebo and commercial vaccine groups. Further development and optimization of the prototype formulation are warranted to enhance its protective efficacy.

## Introduction

Porcine circovirus type 2 (PCV2) is a virus of the circovirus genus, and it is considered one of the most important pathogens in the swine industry due to the high number of syndromes and diseases associated with it. The PCV2 virus, which affects growing and fattening piglets, is recognized as the main etiological agent causing Postweaning Multisystemic Wasting Syndrome (PMWS) (1–4). Several retrospective epidemiological studies have shown that PCV2 is a pathogenic agent present in a large part of the world (3). Twenty-five years ago, PCV2 was considered one of the most important pathogens in swine diseases, since it was identified as the main causative agent of wasting syndrome, reproductive diseases, dermopathies and nephropathies, and respiratory diseases (5, 6).

The pigs are considered as natural hosts for PCV2, which remain chronically infected upon infection (6). PCV2 associated disease are multifactorial, requiring additional intrinsic or extrinsic factors to initiate clinical disease (7). The PCV2 genome contain eleven open reading frames (ORFs). ORF1 the most conserved region, encodes replication-associated proteins, while ORF2 encodes the capsid protein (Cap), the major viral antigen that elicits the strongest host immune response. Because ORF2 exhibits genetic variability, it serves as a key determinant for differentiating PCV2 genotypes. Several other ORFs are involved in cytokine and interleukin expression, cell apoptosis, and viral structure formation (7). Based on ORF2 variability, nine genotypes have been identified, among which PCV2a, PCV2b and PCV2d are the most prevalent and widely distributed worldwide (8), accounting for ~16, 46 and 34% reported genotypes, respectively. However, in South America and Chile, the most prevalent genotype is PCV2b (9). Several studies have shown that nearly all farms worldwide are seropositive for PCV2, indicating that the virus is present in all intensive swine production farms (10), where horizontal transmission through the oro-nasal route represents the main route of viral entry, although vertical transmission can also possible, albeit less frequently and with variable occurrence (7). Once the virus enters the host, it infects it primarily targets antigen-presenting cells notably macrophages and dendritic cells, which phagocytize the virus following infection (11). Viral entry into cells occurs through endocytosis, after which virions accumulate in phagolysosomes and form intracytoplasmic inclusion bodies. During this process, viral particles may associate with mitochondria and migrate toward the nucleus. Within the nucleus, virions cluster in intranuclear inclusion bodies, where they replicate and are encapsidated into new virions (7, 11). Upon release into the cytoplasm, newly formed virions aggregate into intracytoplasmic inclusion bodies and exit the cell either via exocytosis or through lytic release, processes that are associated with marked cellular disruption and death (7, 11).

PCV2 impairs the host's immune response compromising the functionality of dendritic cells and macrophages, reducing their ability to present antigens and secrete key cytokines such as IFN-α and TNF-α (12–14). Additionally, PCV2 infection upregulates IL-10 and SOCS3 expression, which suppress proinflammatory responses and support viral replication (12, 14, 15). The virus also promotes the expansion of regulatory T cells (Tregs) via the TGF-β/Smad3 signaling pathway, inducing immune tolerance and impairing antiviral defenses (16). Moreover, Foxp3 has been shown to inhibit viral replication by reducing the ATPase activity of the viral Rep protein (17).

Following infection, macrophages and dendritic cells disseminate the virus through the lymphoid tissue, infiltrating and displacing resident lymphocytes and serving as the main vehicles system spread (12, 18). Infected mononuclear cells transport the virus via blood to peripheral organs such as lungs, kidneys and intestines where it replicates in epithelial cells, producing lesions like pneumonia, nephritis and enteritis (8, 19). Clinical disease grouped under PCVAD, requires cofactors such as genetic susceptibility or co-infections, notably with porcine parvovirus (PPV), which enhance immune activation and viral load (13, 15, 18).

Subclinical infections detected mainly by serology are the most frequent presentation, affecting 70%−96% of infected pigs, with no evident gross lesions and mild lymphoid depletion microscopically; viral load is typically below 1 × 10^7^ viral particles/ml (20, 21). Among clinical forms, PMWS or PCV2-SD is the most common, with morbidity of 4%−30% and mortality of 4%−20% (20). It is characterized by weight loss, pale skin, respiratory distress, and lymphadenopathy, with histologic findings of granulomatous inflammation, cytoplasmic inclusions, and lymphoid depletion (18, 20, 22). Lung lesions include interstitial pneumonia and necrotizing bronchiolitis, while spleen and liver may show vasculitis, infarcts, or fibrosis; in rare cases, brain lesions with necrotizing vasculitis occur (1, 20, 23, 24).

PCV2 lung disease (PCV2-LD) and enteric disease (PCV2-ED) manifest as respiratory distress and diarrhea, respectively with bronchointerstitial pneumonia or granulomatous enteritis affecting Peyer's patches (1, 20). Quantitative PCR is the preferred diagnostic tool for early detection (25). Vaccination remains the main preventive strategy, most based on the PCV2a genotype and targeting the Cap protein (pCap) (26, 27). Despite high efficacy, occasional clinical cases occur in vaccinated pigs (9). According to the World Organization for Animal Health (WOAH) (28), five commercially vaccines are available worldwide: one inactivated vaccine, three Cap -derived, and one chimeric formulation.

Vaccines against porcine circovirus infection aim to prevent the appearance of severe macroscopic and microscopic lesions. A prototype vaccine was developed based on chimeric antigens derived from the PCV2b genotype, with the aim of providing effective protection against PCV2 infection (29). In this context, the present study aimed to evaluate the effect of this novel vaccine prototype on PCV2-associated histopathological lesions in selected tissues and to assess its correlation with the viral load detected in the vaccinated animals.

## Materials and methods

### Animal selection and experimental design

Piglets used in this study were obtained at weaning age (21 days of age), coming from a high-health-status intensive production farm free of major, globally recognized swine diseases. The animals showed no clinical or subclinical signs of illness at time of acquisition. Piglets were raised under controlled experimental conditions as a part of a study evaluating a chimeric PCV2 experimental vaccine developed within the framework of the FONDEF IT21I0001 project. The trial was conducted at the experimental unit of the Center of Biotechnology and Biomedicine Spa in Quinchamalí, Chile.

The facilities (Supplementary Figure 1) comprised three rooms with a personnel area divides into a dirty zone equipped with a footbath, a shower transition zone, and a clean area containing biosafety equipment. Adjacent to the main room were dedicated storage areas for feed and materials. The animal handling area consisted of three pens (each measuring 12 m^2^), equipped with Rotecna^®^ plastic slap flooring, Cristal Spring^®^ feeders, and *ad libitum* access to drinking water via nipple drinkers. Each pen contained a wooden stall (200 × 90 × 80 cm) with a rubber floor, infrared heating (175 W InterHead 3G infrared bulb), and a thermo-hygrometer (HRC-2) to monitor environmental and stall temperature. The wooden stall was used only during the first weeks of the experiment and was subsequently then removed.

Upon arrival, pigs were allowed a 4-day acclimatization period before trial initiation (day 0). The selection criteria included 21-day-old PIC terminal hybrids piglets of both sexes, clinically healthy, PCV2 by ELISA, and qPCR-negative. All piglets were unvaccinated against PCV2 prior to the experiment.

Body weight was recorded at three specific time points: at the start of the study (day 0), after 7 weeks, and at 17 weeks (previous euthanasia). The weights were used to monitor growth performance and to ensure the general health status of the animals throughout the study period. Clinical signs, including liveliness, respiratory distress, and anorexia, were observed daily to detect any deviation from normal behavior or signs of disease.

**Figure 1:**
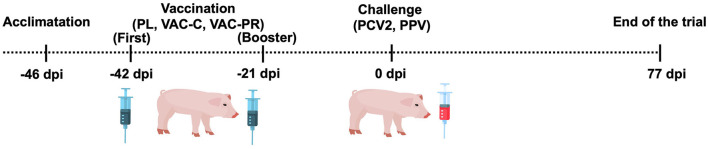
Timeline of the experimental design used in the trial.

Animals were fed *ad libitum* with phase-specific diets formulated for intensive swine production and were randomly divided into three groups (*n* = 13 each): placebo group (PL), receiving an intramuscular placebo solution; commercial vaccine group (VAC-C), administered an inactivated aqueous-based PCV2 vaccine containing the VQ2610 strain (1 × 10^9^ genome copies); and prototype vaccine group (VAC-PR), receiving the oil-based recombinant chimeric vaccine developed by Lamazares et al. (29), formulated with the Qm1 gene and Montanide ISA 50 V2 adjuvant (SEPPIC, Paris, France). All groups received a first intramuscular dose on day 4 post-acclimatization (~25 days of age) and a booster dose 3 weeks later (Figure 1).

Three weeks after booster [day 0 post-inoculation (dpi)], all animals were challenged with PCV2b by intranasal (2 ml) and intramuscular (1 ml) routes at a concentration of 4.3 × 10^7^ viral particles/ml, as described by Segalés (14). This was combined with 1 ml of porcine parvovirus (PPV) diluted 1:50 (cycle threshold = 28) provided by Dr. Victor Neira (University of Chile), as described by Reséndiz et al. (21). Animals remained in the facility until the end of the trial (11.3 weeks post-inoculation), approximating slaughter age.

### Serological analysis

Specific antibodies against PCV2 were detected using INgezim^®^ Circo IgG 11.PCV.K1 ELISSA test (Ingenasa Laboratories, Madrid, Spain) following the manufacturer's instructions. Animal were tested upon arrival and at the end of vaccination period to evaluate seroconversion.

### Sample collection and histopathological evaluation

Pigs were necropsied according the protocol described by Segalés and Domingo (30). Euthanasia was performed with an intravenous overdose of sodium thiopental (5%, 100 mg/kg), administered through the jugular vein, in accordance with American Veterinary Medical Association (AVMA) guidelines (31). Macroscopic lesions were described, and tissue samples from lungs and mediastinal lymph nodes were collected for histopathological analysis. Blood samples were obtained prior to necropsy to assess PCV2 viral load.

### Tissue samples

For histopathological analysis, tissue samples were fixed in 10% neutral buffered formalin (NBF) and routinely processed embedding into paraffin wax and subsequent histopathology according to the standard protocol established by Luna (32). After this, sections were stained with hematoxylin and eosin (H&E) following the standard protocols of the diagnostic laboratory. Slides were examined using an Axioskop 40 microscope equipped with an integrate Canon 1200 camera.

### Viral load quantification by real-time PCR

To determine the viral load in blood, serum samples were used, and DNA extraction was performed using the commercial QIAamp^®^ DNA Mini Kit (Qiagen, Germany), following the manufacturer's instructions. Semi-quantitative real-time PCR was performed using SensiMix™ SYBR^®^ Hi-ROX Kit and oligonucleotides described by Opriessnig et al. (33) (forward: 5′-TGGCCCGCAGTATTCTGATT-3′; reverse: 5′-CAGCTGGGACAGCAGTTGAG-3′). Reactions were run on ABI Prism Primer Express software (V1.5, PE Applied Biosystems, Foster City, CA, USA). Each 50 μl reaction consisted of 25 μl of SensiMix™ SYBR^®^, 0.5 μl of each primer, 5 μl of template DNA, and 19 μl of nuclease-free water. A standard curve was constructed using five 10-fold serial dilutions of PCV2 genomic DNA, starting at 1 × 10^8^ copies, and viral load was expressed as genome copies per ml of serum.

Samples with Ct value corresponding to viral loads ≤ 1.0 × 10^5^ copies/ml were considered negative for PCV2 infection, while values ≥ 1.0 × 10^6^ copies/ml were considered positive (33).

### Macroscopic and microscopic lesions classification

Macroscopic lesions associated with PCV2 focused on enlargement and hyperemia of mediastinal lymph nodes, which were scored as follows: (0 = absent, 1 = mild, 2 = moderate, 3 = severe). Lungs were assessed for collapse and edema.

Microscopic lesions were evaluated according to the criteria described by Segalés (20) and Segalés et al. (34). In lymphoid tissues, lymphoid depletion and granulomatous infiltrate were scored based on lesion severity: 0 = none, 1 = mild, 2 = moderate, 3 = severe. Presence of multinucleated giant cells (MGCs) was recorded as 0 = absent, 1 = present. In lung tissues, septal thickening and peribronchiolar/peribronchial (BALT) infiltrates were similarly scored from 0 to 3 (0 = none, 1 = mild, 2 = moderate, 3 = severe) according to the extent of the lesion.

### Statistics analysis

Sample size was calculated using log_10_-transformed viral load data to account for the log-normal distribution typical of qPCR results. The estimation was performed at 95% confidence level, resulting in 13 animals per experimental group (35).

Differences in mean viral load between groups were analyzed using a paired samples *t*-test for repeated measures, applying a 95% confidence level. Associations between categorical variables, as defined in the lesion scoring tables, were evaluated using the chi-square test or Fisher's exact test when expected frequencies were below five. A 95% confidence level was applied for all analyses (36). The association between viral load and lesions severity was assessed using Fisher's exact test (36).

To assess statistical significance, GraphPad Prism version 10.3.0 (217) for windows (GraphPad Software, LLC) was used for graphical representations and statistical analyses. Normality was evaluated using the D'Agostino and Pearson omnibus normality test. Since data did not follow a normal distribution, statistical differences were assessed by the Kruskal-Wallis nonparametric test. A *p*-value below 0.05 was considered statistically significant and was indicated as follows: with *p* ≤ 0.05 (^*^), *p* ≤ 0.01 (^**^), and *p* ≤ 0.001 (^***^).

## Results

### Body weight gain

**Figure 2:**
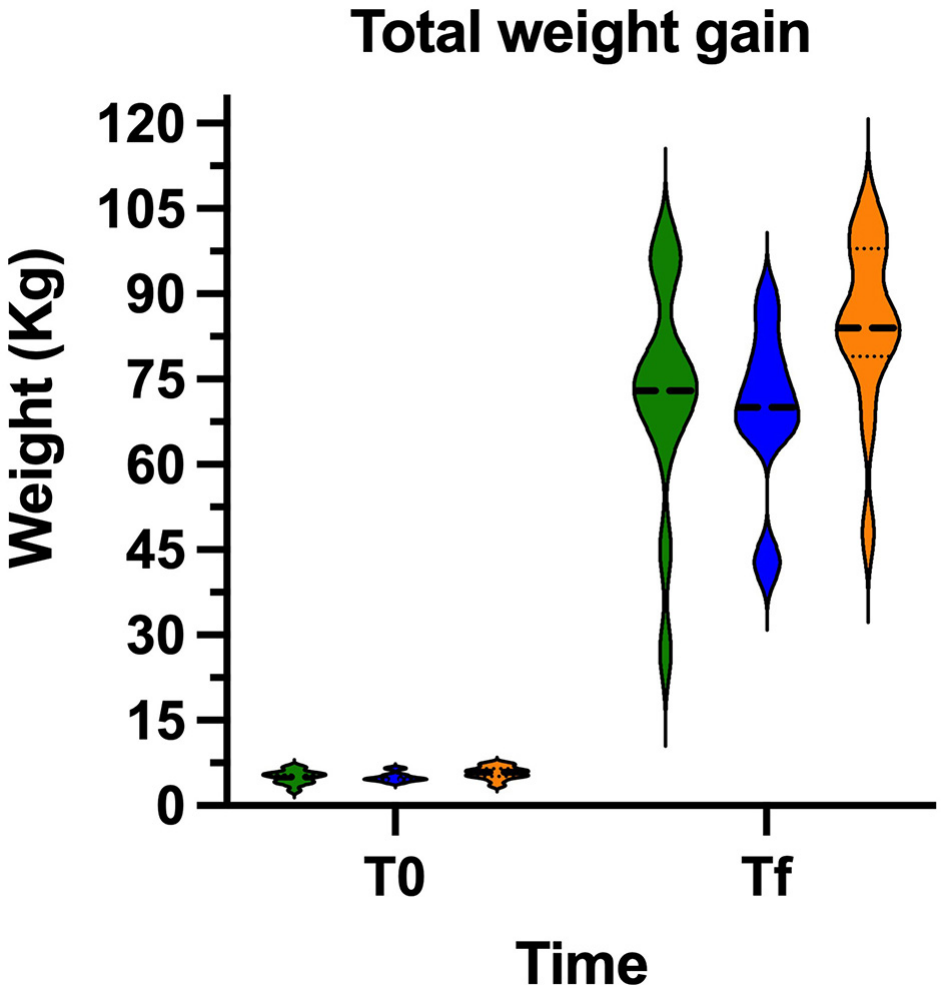
Average weight gain in pigs from the three experimental groups throughout the trial. PL (green), VAC-C (blue), and VAC-P (orange) groups. No statistical differences (*p* > 0.05).

At the time of challenge, all animals appeared clinically healthy. Body weight gain was evaluated to determine the potential impact of vaccination on growth performance during the experimental period. The three experimental groups started the trial with similar mean initial body weights (*p* > 0.05). At the end of the trial, both vaccinated groups showed higher average weight gains compared with the placebo group (Figure 2). Notably, pigs in the VAC-PR group exhibited a marked improvement in weight gain, comparable to that of the VAC-C group. In contrast, the Placebo (PL) group showed the lowest weight gain across the trial, although these differences were not statistical significance (*p* > 0.05).

### Viral load

**Figure 3:**
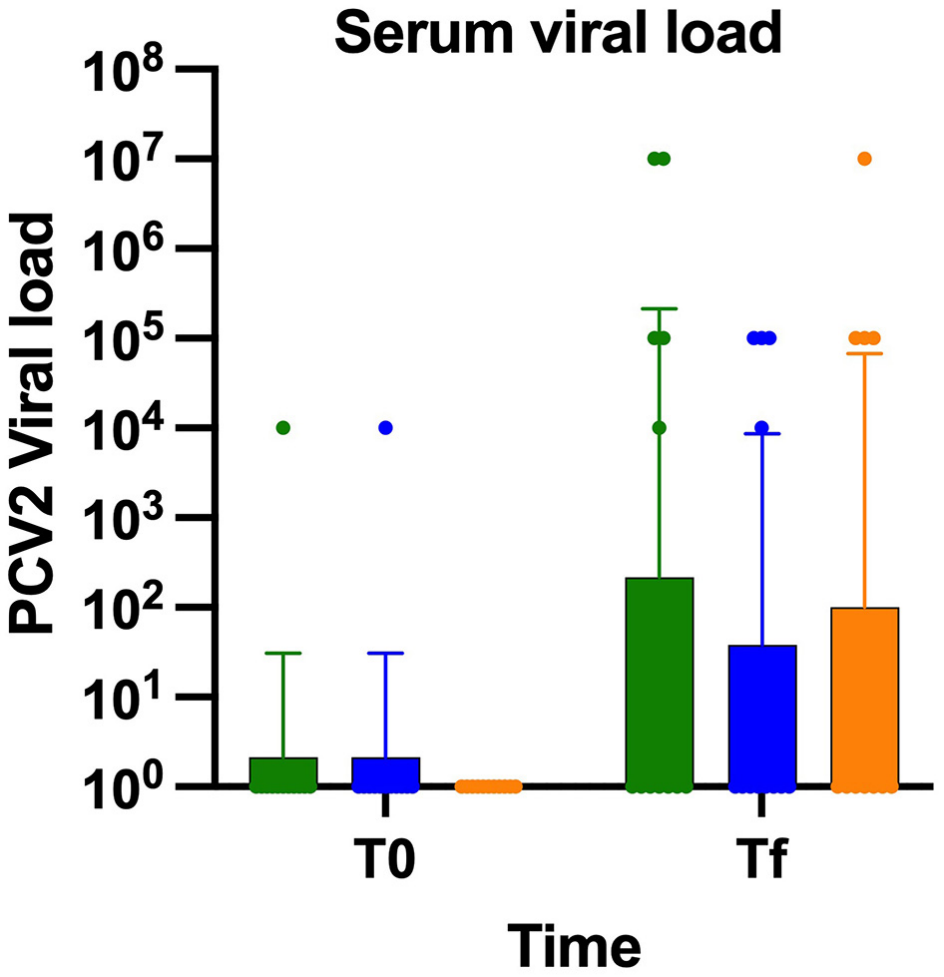
PCV2 serum viral load was quantified in PL (green), VAC-C (blue), and VAC-P (orange) groups. No statistical differences (*p* > 0.05).

To determine the development of viremia, blood samples were taken from the day of challenge until the end of the experiment and analyzed by qPCR viral loads ≤ 1.0 × 10^5^ copies/ml were considered negative, whereas values ≥ 1.0 × 10^6^ copies/ml were classified positive. All animals tested negative by qPCR at day 0. At the end of trial, most animals across the three experimental groups showed no detectable viral genome copies in serum (Table 1). However, two pigs from PL group (1.0 × 10^7^ copies/ml) and one from VAC-PR group (1.0 × 10^7^ copies/ml) presented viral genome copies compatible with infection, while no viral genomes were detected in pigs VAC-C group. No statistically significant differences were observed among the three groups in regarding blood viral loads (*p* > 0.1; Figure 3).

**Table 1:** Results of blood viral load of porcine circovirus type 2 per ml of serum observed by real-time polymerase chain reaction for each experimental group and the end of trial.

**Group**	**Blood viral load/ml**			
	**No Ct**	≤ **10**^5^	**10** ^6^	≥**10**^7^
PL	7	4	0	2
VAC-C	9	4	0	0
VAC-PR	8	4	0	1

Ct, threshold cycle; PL, placebo group; VAC-C, commercial vaccine group; VAC-PR, prototype vaccine group.

### Lesions in the lungs and lymph nodes

Macroscopic examination revealed moderate lymphadenopathy of the mediastinal lymph nodes in the PL group. This same lesion was also identified in both vaccinated groups, though with lower intensity. In contrast, no apparent pulmonary lesions were observed either vaccinated groups. However, animals from the PL group exhibited pulmonary congestion and edema in 50% of the cases.

**Figure 4:**
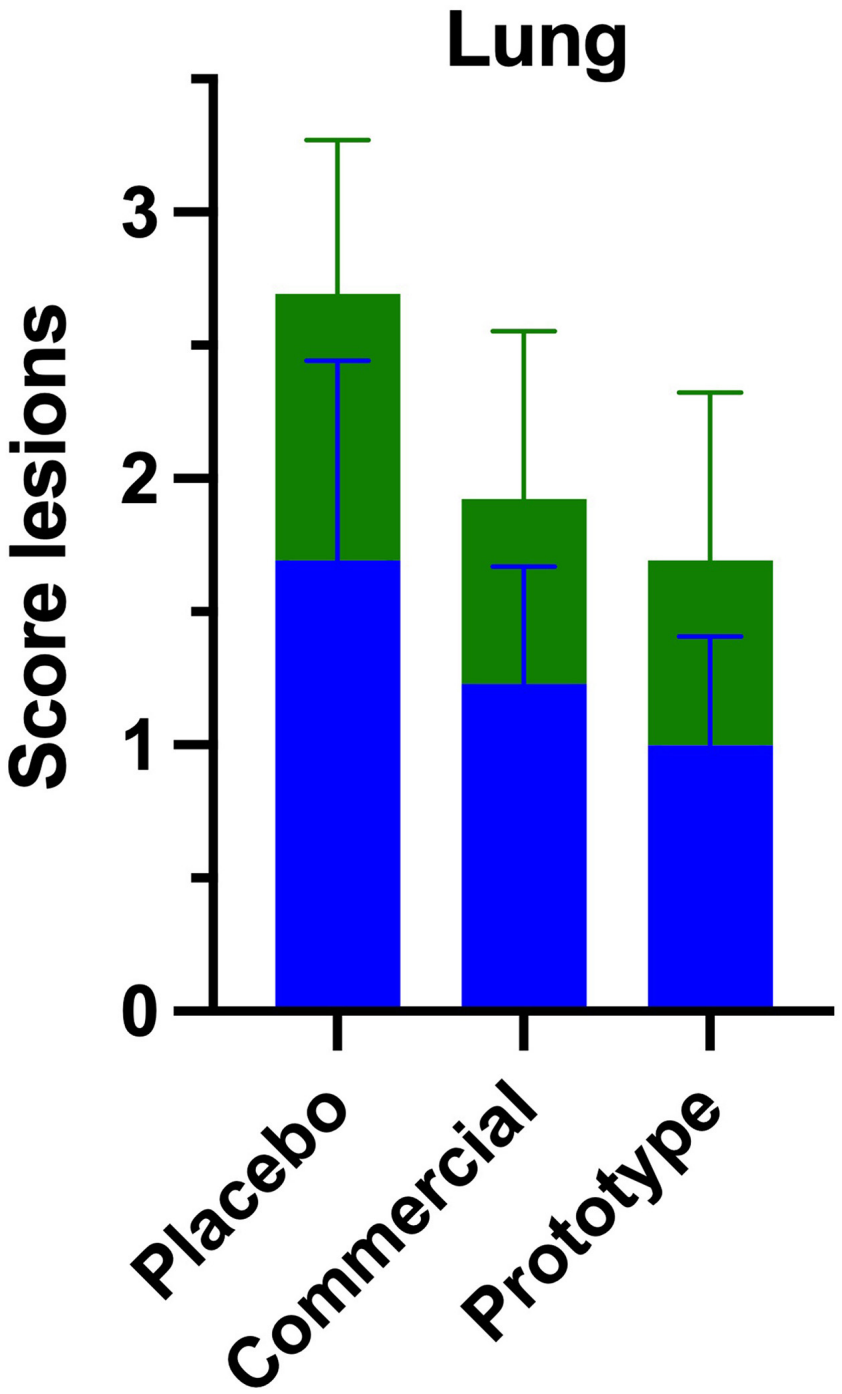
Histopathological scoring lesion in lungs. Mean severity scores for each experimental group, were graded as 0 (absent), 1 (mild), 2 (moderate), and 3 (severe). Representative features include thickening of alveolar septa (green) and peribronchial inflammatory infiltrate (blue).

**Figure 5:**
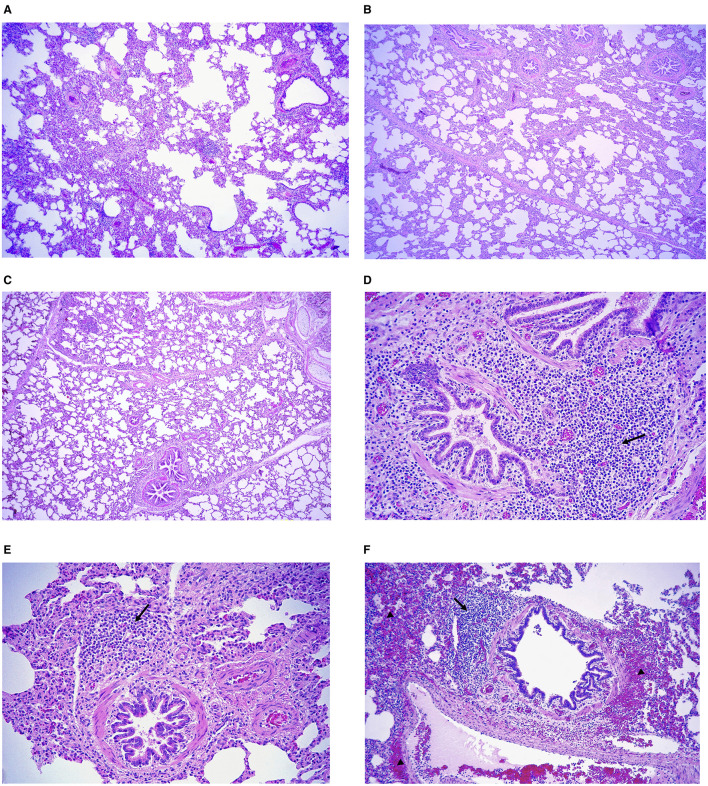
**(A–C)** Microscopic lesion severity in the lungs. Representative interstitial septal thickening in animals from the **(A)** placebo group, **(B)** commercial vaccine, and **(C)** prototype vaccine group. Hematoxylin and eosin. Scale bars: 200 μm. Representative peribronchiolar inflammatory infiltrates (arrows) from animals in the **(D)** placebo group, **(E)** commercial vaccine and **(F)** prototype vaccine group with hemorrhages (arrowhead). Hematoxylin and eosin. Scale bars: 50 μm.

Microscopically lung section from all three experimental groups (Figure 4) showed mild thickening of the alveolar septa, mainly due to mononuclear inflammatory infiltrates composed predominantly of macrophages, lymphocytes, and plasma cells (Figures 5A–C). Macrophages were the most abundant cell type, frequently located within the alveolar septa and peribronchiolar regions. Lymphocytes were distributed diffusely throughout the interstitial and perivascular areas, while peribronchial and peribronchiolar regions exhibited hyperplasia of bronchus-associated lymphoid tissue (BALT), characterized by lymphoid follicle formation with prominent germinal centers (Figures 5D–F). The PL group showed the most severe lesions, characterized by diffuse alveolar septal thickening, congestion, and accumulation of mononuclear cells within the peribronchial and perivascular regions. In contrast, lungs from VAC-C and VAC-PR groups showed lower histological changes, consisting of focal to multifocal interstitial thickening with scant peribronchiolar mononuclear infiltrates and minimal congestion.

Overall, the lesions were less extensive and less intense in both vaccinated groups compared with the PL group, indicating a protective effect of vaccination on the pulmonary parenchyma. No evidence of suppurative bronchopneumonia, vasculitis, or secondary bacterial infection was detected in any group.

**Figure 6:**
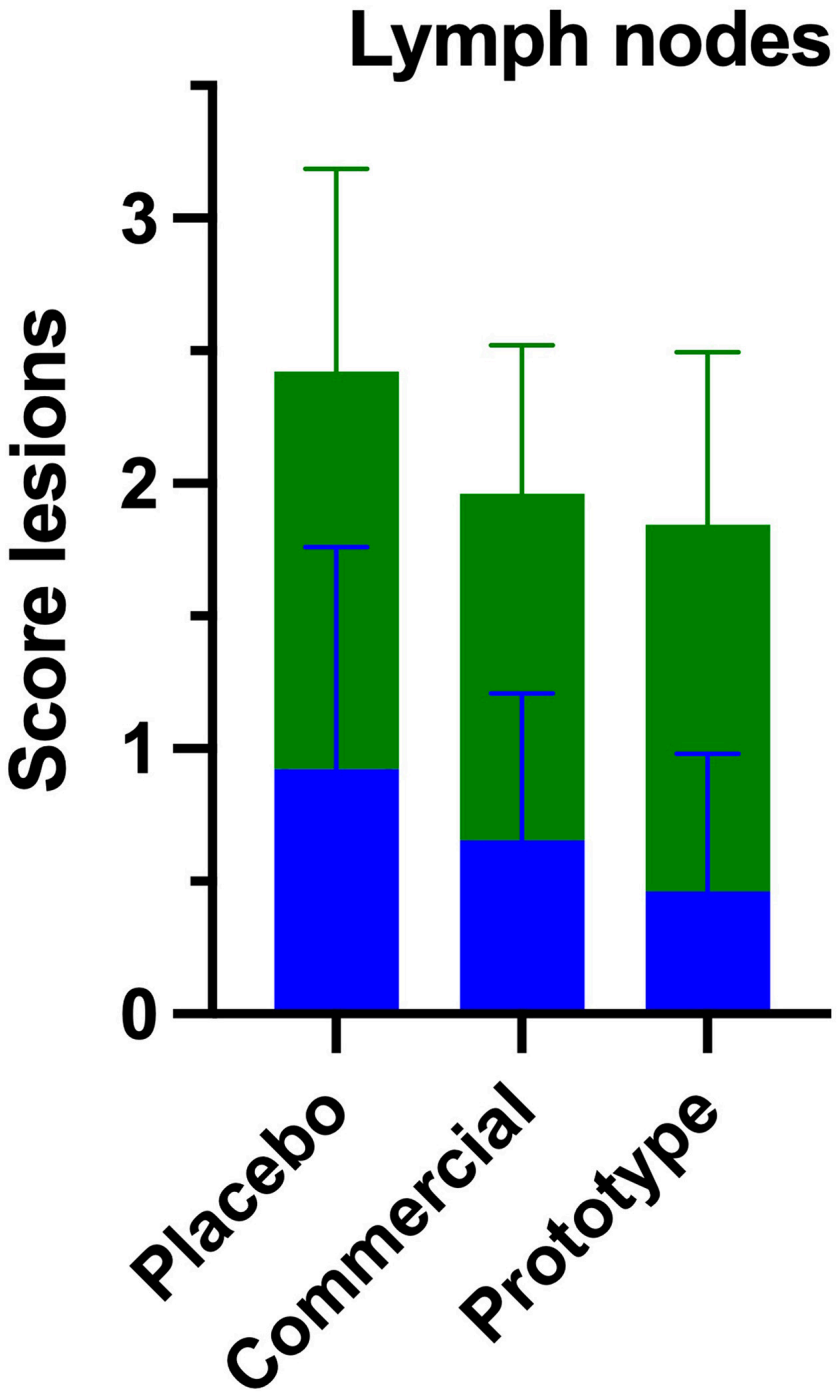
Histopathological scoring of lesion in mediastinal lymph nodes. Mean severity scores for each experimental group were graded as 0 (absent), 1 (mild), 2 (moderate), and 3 (severe). Representative features include histiocytic inflammatory infiltrate (green) and lymphoid depletion (blue).

**Figure 7:**
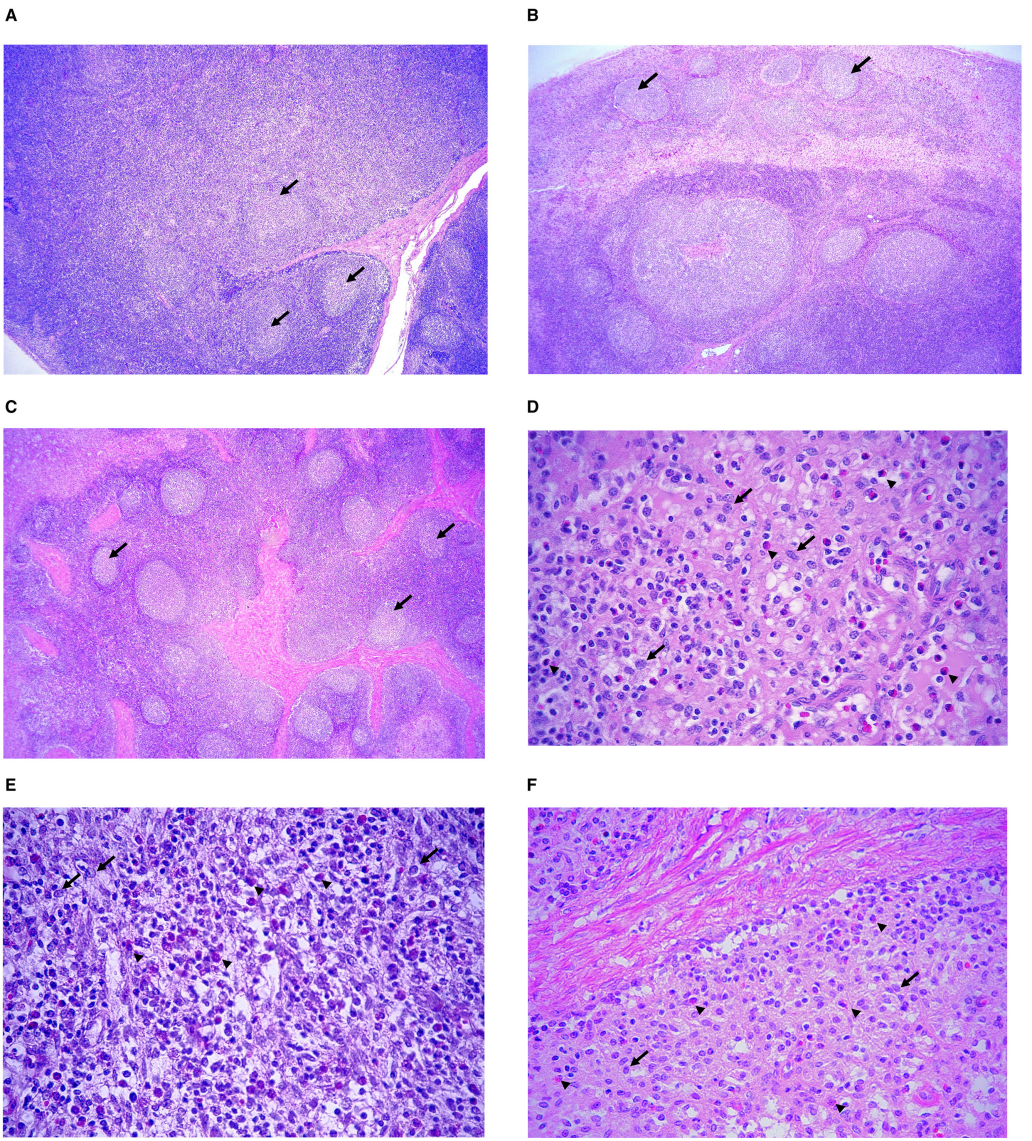
Representative histopathological features in mediastinal lymph nodes. **(A–C)** lymphoid depletion (arrows) in pigs from the **(A)** placebo, **(B)** commercial vaccine and **(C)** prototype vaccine groups. The lymphoid depletion was more pronounced in the placebo group with few lymphoid follicles (arrows), whereas lymphoid architecture was better preserved in the prototype vaccine group (arrows). Hematoxylin and eosin. Scale bars: 200 μm. **(D–F)** Histiocytic infiltration (arrows) in with scattered eosinophilic infiltration (arrowheads) in **(D)** placebo, **(E)** commercial vaccine, and **(F)** prototype vaccine groups. Hematoxylin and eosin. Scale bars: 10 μm.

In all three experimental groups, lymphoid depletion in the lymph nodes was generally mild (Figure 6). However, the PL group exhibited a higher number of animals with moderate to marked lymphoid depletion, particularly affecting the cortical and paracortical regions (Figures 7A–C). In these animals, loss follicular architecture and reduction of germinal centers were frequently observed.

A clear difference was also noted in the peritrabecular and subcapsular histiocytic infiltrates between groups. The PL group (Figure 7D) showed dense accumulations of histiocytes, often accompanied by MGCs within the parafollicular zone, consistent with the granulomatous pattern commonly associated with PCV2 infection. In contrast, both vaccinated groups (VAC-C and VAC-PR) exhibited milder histiocytic infiltration, limited mainly to the subcapsular sinus (Figures 7E, F).

Interestingly, in the VAC-C group, the histiocytic infiltrate frequently contained numerous eosinophils interspersed among mononuclear cells (Figure 7E). The VAC-PR group showed comparable findings but with fewer eosinophils and less disruption of lymphoid structure (Figure 7F).

Overall, the lymphoid depletion and histiocytic reaction were more pronounced in the PL group, while vaccinated animals maintained better follicular organization, indicating a protective effect of vaccination on lymphoid integrity and immune homeostasis.

## Discussion

Effective control of PCV2 requires a multifaceted approach, with vaccines playing a critical role. Despite the widespread use of commercial vaccines, sporadic cases of infection and variable immune responses continue to be reported, emphasizing the need to better understand vaccine efficacy and host–virus interactions under experimental conditions.

In this study, we evaluated the efficacy of a prototype intramuscular PCV2 vaccine in pigs experimentally challenged with a virulent PCV2 strain. The analysis focused on virological and histopathological parameters to determine the level of protection induced by the prototype formulation in comparison with a commercial vaccine and a placebo control. Previous studies have reported that between 70 and 96% of infected animals do not develop clinical disease or show detectable viral loads in the blood, nor do they present macroscopic lesions characteristic with PMWS (20). Therefore, it was expected that 70%−90% of the animals in the trial would not display detectable viral loads or lesions compatible with the disease, which was confirmed by the study results. Indeed, at least 50% of the pigs across all experimental groups did not exhibit viral loads >10^5^ copies/ml or show severe lesions associated with PMWS.

In seven animals of the PL group, no viral replication was detected. It is hypothesized that viremia did not persist in these pigs due to two main factors. First, the expected morbidity of this disease which not exceed 40% even under experimental conditions (13, 14, 19, 20) and second, the adequate dietary supplementation provided throughout the trial, which may have contributed to an effective immune response against infected cells, ultimately leading to a reduction in blood viral load by the end of the study. Zhai et al. (37) reported that oxidative stress enhances viral replication in leukocytes and promotes PCV2 viremia, whereas antioxidant compounds reduce oxidative stress and suppress viral replication. Therefore, a nutrient rich diet high in antioxidants may play a critical role in controlling viral persistence and limiting PCV2-induced damage in vaccinated pigs.

Regarding the VAC-C group, it was expected that they would not replicate the viral genome or that they would be presented a viral load equal to or < 10^5^ copies/ml, as vaccination reduces the amount of virus circulating in the organism. Accordingly, a notable reduction in the intensity of macroscopic and histological lesions was observed, primarily in the lymph nodes and lungs, indicating that the VAC-C vaccine was highly effective in reducing PCV2-associated lesions. Interestingly, a predominance of eosinophilic granulomatous inflammation was observed in the lymph nodes, macroscopically evidenced by enlarged mediastinal lymph nodes. This finding suggests a differential local immune activation pattern rather than a pathological hypersensitivity reaction. Although the exact mechanism remains to be elucidated, it could reflect a Th2-skewed immune response, possibly influenced by the adjuvant composition of the commercial vaccine (38–41).

This interpretation should be regarded as preliminary, as no direct immunophenotypic or cytokine analyses were performed to confirm eosinophil recruitment pathways. Therefore, while a mild hypersensitivity-type reaction cannot be entirely ruled out, the absence of clinical manifestations consistent with hypersensitivity in vaccinated pigs supports a non-adverse immunomodulatory origin (42, 43).

Further studies are warranted to characterize the immunological polarity of this eosinophilic response, including the quantification of Th2 cytokines such as IL-4, IL-5, IL-13 and histochemical markers of eosinophil activation, to clarify whether this phenomenon represents an adjuvant-induced bias or a beneficial immunoregulatory feature of the commercial formulation (41, 44).

According to Zachary (45), the chronic phase of a type I hypersensitivity reaction is responsible for the recruitment of eosinophils, a mechanism observed in this experimental group. Moreover, this response is usually focal, and its severity depends on the individual's susceptibility to histamine, which, in this case, did not trigger an intense reaction. On the other hand, Batista-Duharte et al. (46) indicate that certain types of adjuvants can cause local adverse side effects, including delayed recruitment of immune cells that release proinflammatory cytokines, resulting in a delayed hypersensitivity reaction. It is hypothesized that the adjuvants present in the commercial vaccine may have induced the eosinophilic infiltrate observed in the lymph nodes, consistent with a local and mild hypersensitivity reaction.

The results show that VAC-PR pig group presented intermediate outcomes between the PL and VAC-C groups. Their viral loads, as well as their macroscopic and histological lesions, were comparable to those observed in the VAC-C group. However, one pig in the VAC-PR group showed a viral genome copy number consistent with clinical PCV2 infection, although its lesions resembled those of the subclinical form of the disease. These results suggest that the VAC-PR provides partial protection against viremia; however, since it is still under development, its performance is not yet fully comparable to that of a VAC-C, as some animals showed blood viral loads indicative of the disease. According to Batista-Duharte et al. (46), adjuvants contribute significantly to the potency, quality, and duration of the immune response. In this context, it is presumed that VAC-C achieved a stronger response against blood viral loads and tissue lesions because its aqueous-based adjuvants were specifically designed to optimize its efficacy, whereas VAC-PR used commercial oil-based adjuvants.

Regarding to this, although Porcine parvovirus (PPV) is recognized as a potential cofactor that can enhance the replication and pathogenicity of PCV2 during the acute phase of infection (47), the present study demonstrated that both the experimental chimeric vaccine (VAC-PR) and the commercial vaccine (VAC-C) provided effective protection against the deleterious effects typically associated with such co-infections. Both vaccinated groups exhibited reduced viremia and milder microscopic lesions compared with the unvaccinated group, indicating that vaccination could mitigate the synergistic impact usually observed between PPV and PCV2.

Importantly, no adverse systemic reactions or clinical signs suggestive of vaccine-induced hypersensitivity or exacerbated pathology were recorded, supporting the safety and tolerability of both formulations under co-infection conditions. This contrasts with classical experimental co-infection models, where unprotected pigs developed pronounced lymphoid depletion, pulmonary inflammation, and elevated viral loads (47, 48). The absence of these effects in vaccinated animals strongly suggests that immunization effectively modulated the host immune response, preventing the amplification of PCV2 replication typically triggered by PPV-induced immunomodulation. Therefore, the results of this study indicate that both vaccines were effective in reducing PCV2 replication under experimental challenge conditions.

The lack of statistically significance differences in viral loads between the vaccinated groups and the PL group is likely due to the challenges of maintaining sustained viral replication under experimental conditions over an extended period. This limitation hindered the successful replication of infection until the end of the trial in most of the animals in the PL group, as previously reported by Segalés (20). Nevertheless, a notable reduction in viral load was observed in the vaccinated groups compared to the unvaccinated group, indicating a trend toward viral control associated with vaccination. Although no statistically significant differences in weight gain were detected among the groups, vaccinated animals, in special VAC-PR group, showed a higher average weight gain compared to the unvaccinated group. This tendency suggests that both vaccines may help reduce the productive impact of PCV2 infection, with the prototype vaccine demonstrating promising performance. These findings support the potential of the VAC-PR candidate as an effective alternative for improving both health and productivity outcomes in swine. Additionally, while several animals with high viral loads were identified in the PL group, only one pig in the VAC-PR group exhibited a high viral load. These findings confirm that viral replication persisted until the end of the trial in those specific cases.

## Conclusion

The findings of this study demonstrate that the VAC-PR provided partial protection against PCV2-associated viremia and histopathological damage. Although VAC-PR showed promise in reducing the histopathological impact of PCV2, its protective efficacy was slightly lower than that of the commercial vaccine, likely due to differences in adjuvant formulation. This suggests that further optimization of the adjuvant system or antigen design may enhance the immunogenicity and consistency of the prototype vaccine.

## Data Availability

The original contributions presented in the study are included in the article/Supplementary material, further inquiries can be directed to the corresponding author.

## Supplementary material

Supplementary Figure 1 Layout of the facilities. Entry to the premises through a footbath (red arrow), followed by a transition area equipped with a shower and biosafety gear, which provides access to the main room (blue arrow). (1) To the right, a nursing room containing disposable materials. (2) At the end of the corridor, a feed storage room. (3) To the left, three pig pens, each measuring 12 m^2^.
