# Supplementary figures and images for: Assessment of a porcine circovirus type 2 vaccine prototype through anatomopathological analysis and its correlation with blood viral load
Source: Front Vet Sci. 2026 Jan 2;12:1656345. doi: 10.3389/fvets.2025.1656345 (PMC12807907; doi:10.3389/fvets.2025.1656345)

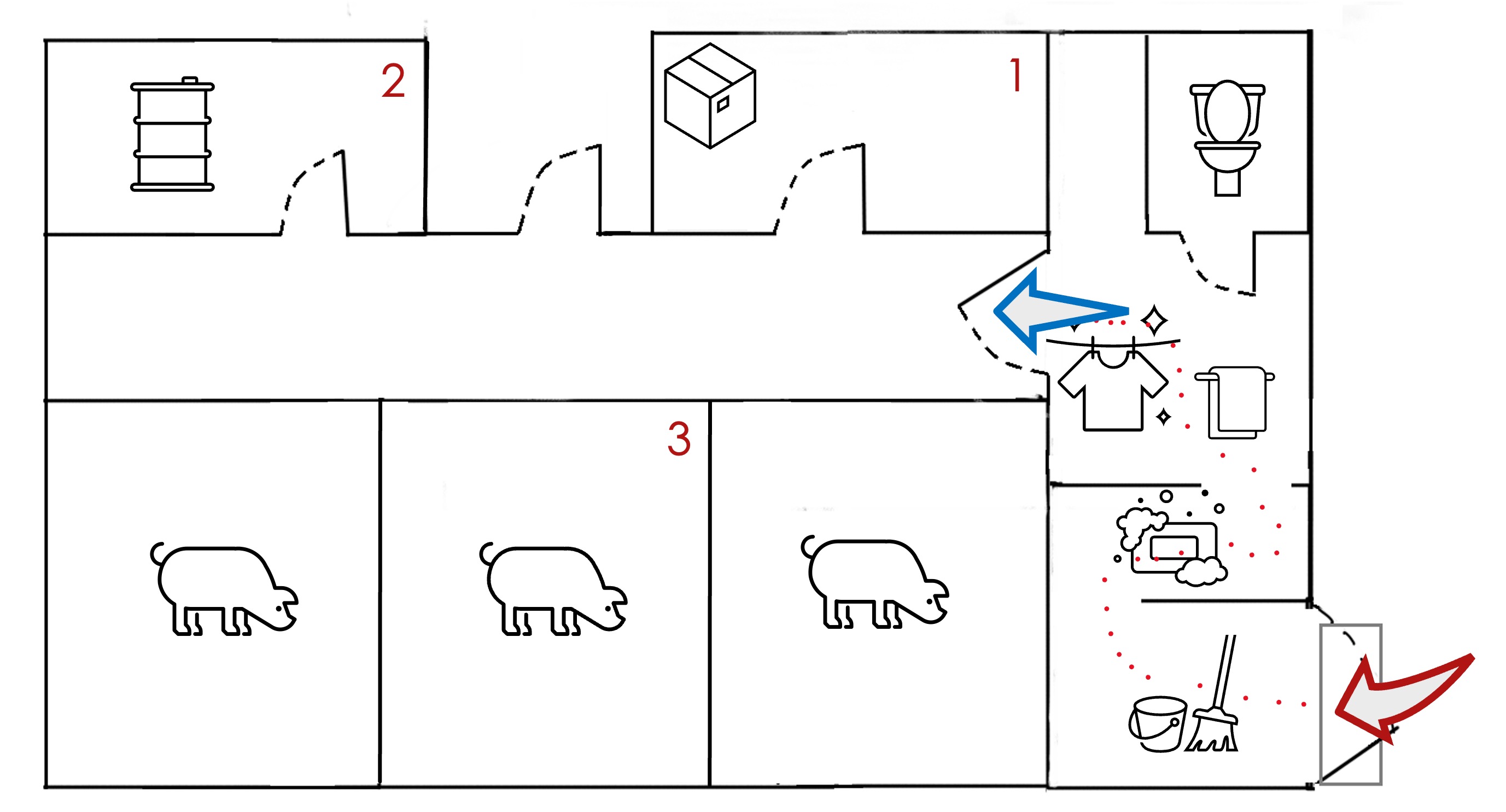

Supplement: Supplementary Figure 1 — Layout of the facilities. Entry to the premises through a footbath (red arrow), followed by a transition area equipped with a shower and biosafety gear, which provides access to the main room (blue arrow). (1) To the right, a nursing room containing disposable materials. (2) At the end of the corridor, a feed storage room. (3) To the left, three pig pens, each measuring 12 m2. [file Image_1.jpeg]
